# 4-Meth­oxy­benzoyl-*meso*-octa­methyl­calix[2]pyrrolidino[2]pyrrole: an acyl chloride derivative of a partially reduced calix[4]pyrrole

**DOI:** 10.1107/S1600536812008008

**Published:** 2012-03-07

**Authors:** Guillaume Journot, Reinhard Neier, Helen Stoeckli-Evans

**Affiliations:** a Institute of Chemistry, University of Neuchâtel, Avenue de Bellevaux 51, CH-2000 Neuchâtel, Switzerland; b Institute of Physics, University of Neuchâtel, rue Emile-Argand 11, CH-2000 Neuchâtel, Switzerland

## Abstract

In the title compound, C_36_H_50_N_4_O_2_, the two pyrrolidine rings have envelope conformations. The conformation of the macrocycle is stabilized by N—H⋯N hydrogen bonds and a C—H⋯N inter­action. The benzoyl ring is inclined to an adjacent pyrrole ring by 6.76 (9)°, with a centroid-to-centroid distance of 3.6285 (10) Å. In the crystal, apart from a C—H⋯O and a C—H⋯π inter­action, mol­ecules are linked *via* an N—H⋯O hydrogen bond, leading to the formation of helical chains propagating along [010].

## Related literature

For the heterogeneous catalytic hydrogenation of *meso*-octa­methyl­calix[4]pyrrole, which gave *meso*-octa­methyl­calix[2]pyrrole­[2]pyrrolidine, see: Blangy *et al.* (2009 ▶). For the *N*-acyl­ation of pyrrolidines using substituted benzoyl chlorides, see: Journot *et al.* (2012*a* ▶); Zhang *et al.* (2009 ▶). For the synthesis and reactivity of the title compound, see: Journot & Neier (2012 ▶). For the crystal structures of similar compounds, see: Journot *et al.* (2012*b* ▶,*c* ▶,*d* ▶,*e* ▶)
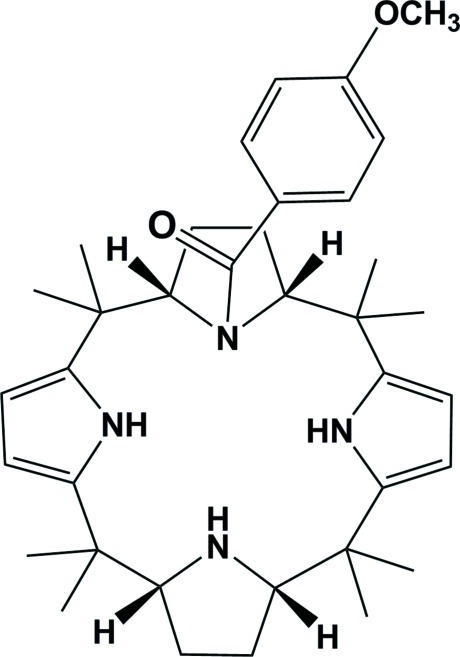


## Experimental

### 

#### Crystal data

C_36_H_50_N_4_O_2_
*M* *_r_* = 570.80
Monoclinic, 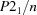

*a* = 10.3150 (4) Å
*b* = 11.8104 (5) Å
*c* = 26.1856 (10) Å
β = 98.629 (3)°
*V* = 3153.9 (2) Å^3^
*Z* = 4
Mo *K*α radiation
μ = 0.08 mm^−1^
*T* = 173 K
0.40 × 0.39 × 0.39 mm

#### Data collection

Stoe IPDS II diffractometer
Absorption correction: multi-scan (*MULABS* in *PLATON*; Spek, 2009 ▶) *T*_min_ = 0.893, *T*_max_ = 1.000
33803 measured reflections
5943 independent reflections
4470 reflections with *I* > 2σ(*I*)
*R*_int_ = 0.063

#### Refinement

*R*[*F* ^2^ > 2σ(*F* ^2^)] = 0.045
*wR*(*F* ^2^) = 0.097
*S* = 1.03
5943 reflections
393 parameters
H atoms treated by a mixture of independent and constrained refinement
Δρ_max_ = 0.20 e Å^−3^
Δρ_min_ = −0.17 e Å^−3^

### 

Data collection: *X-AREA* (Stoe & Cie, 2009 ▶); cell refinement: *X-AREA*; data reduction: *X-RED32* (Stoe & Cie, 2009 ▶); program(s) used to solve structure: *SHELXS97* (Sheldrick, 2008 ▶); program(s) used to refine structure: *SHELXL97* (Sheldrick, 2008 ▶); molecular graphics: *PLATON* (Spek, 2009 ▶); software used to prepare material for publication: *SHELXL97*, *PLATON* and *publCIF* (Westrip, 2010 ▶).

## Supplementary Material

Crystal structure: contains datablock(s) I, global. DOI: 10.1107/S1600536812008008/aa2052sup1.cif

Structure factors: contains datablock(s) I. DOI: 10.1107/S1600536812008008/aa2052Isup2.hkl

Supplementary material file. DOI: 10.1107/S1600536812008008/aa2052Isup3.cml

Additional supplementary materials:  crystallographic information; 3D view; checkCIF report

## Figures and Tables

**Table 1:** Hydrogen-bond geometry (Å, °) *Cg*1 is the centroid of the pyrrole ring N2/C3/C4/C25/C26 and *Cg*2 is the centroid of the benzene ring C30–C35.

*D*—H⋯*A*	*D*—H	H⋯*A*	*D*⋯*A*	*D*—H⋯*A*
N2—H2*A*⋯N3	0.88	2.57	3.0671 (18)	117
N4—H4*A*⋯N3	0.88	2.33	2.8759 (18)	121
C28—H28*B*⋯N4	0.98	2.59	3.561 (2)	171
C28—H28*B*⋯*Cg*1	0.98	2.45	3.3632 (18)	155
N3—H3N⋯O1^i^	0.924 (18)	2.283 (18)	3.1401 (17)	154.0 (15)
C20—H20*B*⋯O1^i^	0.98	2.56	3.530 (2)	170
C15—H15*A*⋯*Cg*2^i^	0.98	2.85	3.7176 (19)	148

Symmetry code: (i) 
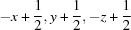
.

**Fig. 1.**
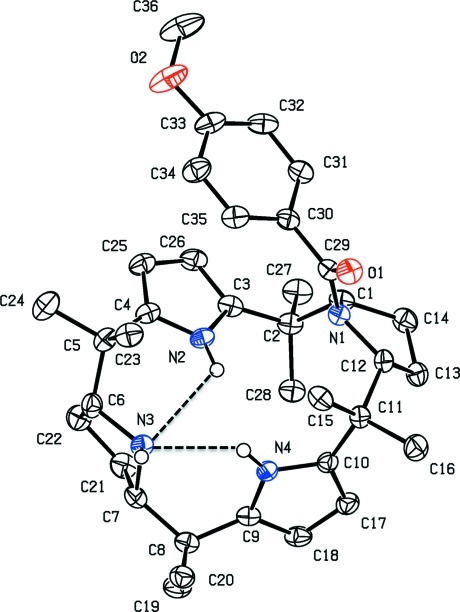
A view of the molecular structure of the title compound, with the numbering scheme and displacement ellipsoids drawn at the 50% probability level. The N—H···N hydrogen bonds are shown as dashed lines (see Table 1 for details; the C-bound H atoms have been omitted for clarity).

**Fig. 2.**
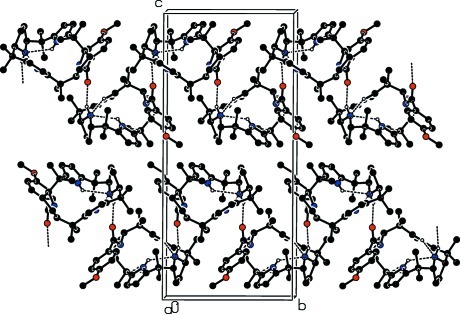
A view along the a axis of the crystal packing of the title compound. The N—H···N and N—H···O hydrogen bonds are shown as dashed lines (see Table 1 for details; the C-bound H atoms have been omitted for clarity).

**Fig. 3.**
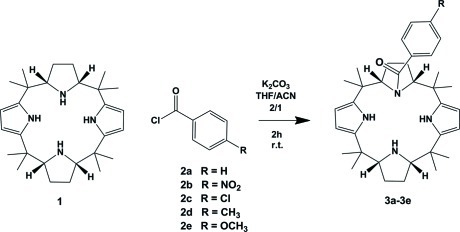
The general procedure for the N-acylation of meso-octamethylcalix[2]pyrrolidino[2]pyrrole (1).

## supplementary crystallographic information

### Comment

We have recently reported the access to new macrocycles by heterogeneous catalytic hydrogenation of *meso*-octamethylcalix[4]pyrrole, which gave *meso*-octamethylcalix[2]pyrrole[2]pyrrolidine (1 in Fig. 3) (Blangy *et al.*, 2009). It was decided to investigate the nucleophilicity of this new macrocycle, which showed interesting reactivity (Journot & Neier, 2012), by reacting different substituted benzoyl chlorides with the macrocycle under smooth conditions (Journot *et al.*, 2012*a*; Zhang *et al.*, 2009). Herein, we report on the synthesis and crystal structure of the title 4-methoxybenzoyl derivative, one of five compounds that have been studied by X-ray diffraction analysis (Journot *et al.*, 2012*b*,*c*,*d*,*e*).

The molecular structure of the title compound is given in Fig. 1. The two pyrrolidine rings (N1,C1,C12–C14) and (N3,C6,C7,C21,C22) have envelope conformations with, respectively, atoms C14 and C7 as the flaps. The conformation of the macrocycle is stabilized by intramolecular N—H···N hydrogen bonds involving atom N3 and the two pyrrole H atoms, H2 and H4 (Table 1). The benzoyl ring (C30–C35) is inclined to the pyrrole ring (N2,C3,C4,C25,C26) by 6.76 (9)°, with a centroid-to-centroid distance of 3.6285 (10) Å. The methyl group C28 is also in close contact with the pyrrole ring (N4,C9,C10,C17,C18), with a short C28—H28A···N4 interaction and a C28—H28A···centroid distance of 3.3632 (18) Å (Table 1).

In the crystal, molecules are linked *via* an N—H···O hydrogen bond, involving the N3 pyrrolidine H atom (H3N) and the benzoyl O atom (O1), leading to the formation of helical chains propagating along [010] (Fig. 2 and Table 1). The same O atom is involved in a C—H···O contact with methyl group C20. A C—H···π interaction is also observed, involving the methyl group C15 and the benzoyl ring (C30–C35) (see Table 1).

The overall geometry and crystal packing is very similar to that reported for the 4-chlorobenzoyl derivative (Journot *et al.*, 2012*b*), and the 4-nitrobenzoyl (Journot *et al.*, 2012*d*) and 4-methylbenzoyl (Journot *et al.*, 2012*e*) derivatives. The benzoyl derivative (Journot *et al.*, 2012*c*) crystallized in the trigonal space group *R*3, as a partial (0.25H_2_O) hydrate, and forms hydrogen bonded chains propagating along [001].

### Experimental

General procedure for the *N*-acylation of *meso*-octamethylcalix[2]pyrrolidino[2]pyrrole (1) is illustrated in Fig. 3. The full details of this synthesis will be reported elsewhere (Journot & Neier, 2012). The title amide 3e was prepared, according to the general procedure, from 100 mg of 1 (0.23 mmol), 4-methoxybenzoyl chloride (2e, 64.93 µl, 0.48 mmol), potassium carbonate (70 mg, 0.48 mmol) in THF (5 ml) and ACN (2.5 ml). The residue was purified by column chromatography (SiO_2_, CH_2_Cl_2_/MeOH, 97/3) to yield 117.0 mg (90%) of colourless crystals of the title compound (3e). Melting point: 488 K. HRMS calcd. for C_36_H_50_N_4_O_2_+H^+^ 571.4007, found 571.3993. Further synthetic and spectroscopic data has been reported elsewhere (Journot & Neier, 2012).

### Refinement

The NH H atoms were located in a difference electron-density map. H atom H3N was freely refined, while the other NH H atoms and the C-bound H atoms were included in calculated positions and treated as riding atoms: N—H = 0.88 Å, C—H = 0.95 Å for CH-allyl and CH-aromatic H atoms, and 1.00, 0.99 and 0.98 Å, for methine, methylene and methyl H atoms, respectively, with *U*_iso_(H) = k × *U*_eq_(C, N), where k = 1.5 for CH_3_ H atoms, and = 1.2 for the other H atoms.

### Crystal data

**Table:**

C_36_H_50_N_4_O_2_	*F*(000) = 1240
*M**_r_* = 570.80	*D*_x_ = 1.202 Mg m^−^^3^
Monoclinic, *P*2_1_/*n*	Melting point: 488 K
Hall symbol: -P 2yn	Mo *K*α radiation, λ = 0.71073 Å
*a* = 10.3150 (4) Å	Cell parameters from 22254 reflections
*b* = 11.8104 (5) Å	θ = 1.6–26.1°
*c* = 26.1856 (10) Å	µ = 0.08 mm^−^^1^
β = 98.629 (3)°	*T* = 173 K
*V* = 3153.9 (2) Å^3^	Block, colourless
*Z* = 4	0.40 × 0.39 × 0.39 mm

### Data collection

**Table:**

Stoe IPDS II diffractometer	5943 independent reflections
Radiation source: fine-focus sealed tube	4470 reflections with *I* > 2σ(*I*)
Graphite monochromator	*R*_int_ = 0.063
φ and ω scans	θ_max_ = 25.6°, θ_min_ = 1.6°
Absorption correction: multi-scan (*MULABS* in *PLATON*; Spek, 2009)	*h* = −12→12
*T*_min_ = 0.893, *T*_max_ = 1.000	*k* = −14→14
33803 measured reflections	*l* = −31→31

### Refinement

**Table:**

Refinement on *F*^2^	Secondary atom site location: difference Fourier map
Least-squares matrix: full	Hydrogen site location: inferred from neighbouring sites
*R*[*F*^2^ > 2σ(*F*^2^)] = 0.045	H atoms treated by a mixture of independent and constrained refinement
*wR*(*F*^2^) = 0.097	*w* = 1/[σ^2^(*F*_o_^2^) + (0.0447*P*)^2^ + 0.4659*P*] where *P* = (*F*_o_^2^ + 2*F*_c_^2^)/3
*S* = 1.03	(Δ/σ)_max_ < 0.001
5943 reflections	Δρ_max_ = 0.20 e Å^−^^3^
393 parameters	Δρ_min_ = −0.17 e Å^−^^3^
0 restraints	Extinction correction: *SHELXL97* (Sheldrick, 2008), Fc^*^=kFc[1+0.001xFc^2^λ^3^/sin(2θ)]^-1/4^
Primary atom site location: structure-invariant direct methods	Extinction coefficient: 0.0016 (3)

### Special details

**Table:**

Geometry. Bond distances, angles *etc*. have been calculated using the rounded fractional coordinates. All su's are estimated from the variances of the (full) variance-covariance matrix. The cell e.s.d.'s are taken into account in the estimation of distances, angles and torsion angles
Refinement. Refinement of *F*^2^ against ALL reflections. The weighted *R*-factor *wR* and goodness of fit *S* are based on *F*^2^, conventional *R*-factors *R* are based on *F*, with *F* set to zero for negative *F*^2^. The threshold expression of *F*^2^ > σ(*F*^2^) is used only for calculating *R*-factors(gt) *etc*. and is not relevant to the choice of reflections for refinement. *R*-factors based on *F*^2^ are statistically about twice as large as those based on *F*, and *R*-factors based on ALL data will be even larger.

### Fractional atomic coordinates and isotropic or equivalent isotropic displacement parameters (Å^2^)

**Table:**

	*x*	*y*	*z*	*U*_iso_*/*U*_eq_	
O1	0.20707 (11)	0.58944 (10)	0.24279 (4)	0.0303 (4)	
O2	0.54533 (13)	0.47589 (14)	0.07206 (6)	0.0546 (5)	
N1	0.04328 (12)	0.64660 (11)	0.17968 (5)	0.0219 (4)	
N2	0.14268 (12)	0.82620 (11)	0.10133 (5)	0.0241 (4)	
N3	0.17847 (13)	1.07140 (11)	0.13942 (5)	0.0240 (4)	
N4	−0.01564 (12)	0.97053 (11)	0.19335 (5)	0.0220 (4)	
C1	−0.03465 (15)	0.60651 (14)	0.13026 (6)	0.0251 (5)	
C2	−0.05119 (16)	0.69079 (14)	0.08382 (6)	0.0258 (5)	
C3	0.08118 (16)	0.73569 (13)	0.07453 (6)	0.0245 (5)	
C4	0.25883 (15)	0.85139 (14)	0.08367 (6)	0.0255 (5)	
C5	0.34568 (16)	0.94944 (15)	0.10384 (6)	0.0282 (5)	
C6	0.26805 (16)	1.06197 (14)	0.10030 (6)	0.0275 (5)	
C7	0.07249 (16)	1.14824 (14)	0.11697 (6)	0.0259 (5)	
C8	−0.04038 (16)	1.16205 (14)	0.14923 (6)	0.0262 (5)	
C9	−0.09460 (15)	1.05203 (13)	0.16707 (6)	0.0245 (5)	
C10	−0.08901 (15)	0.88939 (13)	0.21376 (6)	0.0227 (5)	
C11	−0.02631 (15)	0.79183 (13)	0.24618 (6)	0.0236 (5)	
C12	−0.04177 (15)	0.67167 (14)	0.22005 (6)	0.0233 (5)	
C13	−0.18076 (16)	0.64202 (14)	0.19360 (7)	0.0279 (5)	
C14	−0.16030 (16)	0.56400 (15)	0.14901 (7)	0.0306 (5)	
C15	0.11827 (16)	0.81902 (14)	0.26463 (7)	0.0288 (5)	
C16	−0.09630 (18)	0.78299 (16)	0.29437 (7)	0.0347 (6)	
C17	−0.21771 (16)	0.92137 (14)	0.20026 (7)	0.0283 (5)	
C18	−0.22099 (16)	1.02167 (14)	0.17079 (7)	0.0292 (5)	
C19	−0.15049 (17)	1.22845 (15)	0.11586 (7)	0.0346 (6)	
C20	0.01016 (17)	1.23347 (14)	0.19778 (7)	0.0308 (5)	
C21	0.04096 (17)	1.10045 (15)	0.06251 (6)	0.0316 (6)	
C22	0.17762 (17)	1.08058 (16)	0.04826 (6)	0.0334 (6)	
C23	0.41022 (16)	0.93032 (15)	0.16006 (7)	0.0322 (6)	
C24	0.45482 (18)	0.96246 (18)	0.06999 (7)	0.0414 (7)	
C25	0.27112 (17)	0.77462 (15)	0.04554 (7)	0.0312 (5)	
C26	0.16092 (17)	0.70252 (15)	0.03980 (6)	0.0301 (5)	
C27	−0.10938 (17)	0.61989 (16)	0.03625 (7)	0.0340 (6)	
C28	−0.14476 (16)	0.78916 (14)	0.08942 (6)	0.0288 (5)	
C29	0.16336 (15)	0.59792 (13)	0.19642 (6)	0.0234 (5)	
C30	0.25177 (15)	0.56260 (13)	0.15833 (6)	0.0240 (5)	
C31	0.23655 (17)	0.46683 (14)	0.12715 (6)	0.0294 (5)	
C32	0.33205 (17)	0.43414 (15)	0.09790 (7)	0.0331 (5)	
C33	0.44455 (17)	0.49856 (17)	0.09941 (7)	0.0346 (6)	
C34	0.46181 (17)	0.59379 (16)	0.13061 (7)	0.0360 (6)	
C35	0.36688 (16)	0.62456 (15)	0.15988 (7)	0.0295 (5)	
C36	0.5385 (2)	0.3742 (2)	0.04287 (10)	0.0684 (10)	
H1	0.01140	0.53830	0.11920	0.0300*	
H2A	0.11210	0.86280	0.12620	0.0290*	
H3N	0.2223 (17)	1.0984 (15)	0.1704 (7)	0.031 (5)*	
H4A	0.07050	0.97030	0.19670	0.0260*	
H6	0.33220	1.12590	0.10560	0.0330*	
H7	0.11240	1.22470	0.11410	0.0310*	
H12	−0.01830	0.61490	0.24830	0.0280*	
H13A	−0.23050	0.60290	0.21790	0.0340*	
H13B	−0.22910	0.71110	0.18060	0.0340*	
H14A	−0.23590	0.56850	0.12090	0.0370*	
H14B	−0.14980	0.48450	0.16090	0.0370*	
H15A	0.12560	0.89470	0.28010	0.0430*	
H15B	0.16670	0.81680	0.23520	0.0430*	
H15C	0.15500	0.76290	0.29040	0.0430*	
H16A	−0.18870	0.76360	0.28360	0.0520*	
H16B	−0.09020	0.85580	0.31260	0.0520*	
H16C	−0.05430	0.72400	0.31750	0.0520*	
H17	−0.29180	0.88260	0.20920	0.0340*	
H18	−0.29770	1.06130	0.15610	0.0350*	
H19A	−0.19130	1.18050	0.08740	0.0520*	
H19B	−0.11360	1.29620	0.10190	0.0520*	
H19C	−0.21670	1.25100	0.13710	0.0520*	
H20A	0.03940	1.30760	0.18710	0.0460*	
H20B	0.08370	1.19430	0.21850	0.0460*	
H20C	−0.06070	1.24370	0.21840	0.0460*	
H21A	−0.00950	1.15530	0.03880	0.0380*	
H21B	−0.00900	1.02880	0.06210	0.0380*	
H22A	0.17790	1.01320	0.02580	0.0400*	
H22B	0.20670	1.14710	0.02990	0.0400*	
H23A	0.46920	0.86500	0.16170	0.0480*	
H23B	0.34220	0.91600	0.18170	0.0480*	
H23C	0.46040	0.99780	0.17270	0.0480*	
H24A	0.41530	0.97620	0.03410	0.0620*	
H24B	0.50730	0.89300	0.07190	0.0620*	
H24C	0.51120	1.02650	0.08250	0.0620*	
H25	0.34190	0.77060	0.02620	0.0370*	
H26	0.14470	0.64160	0.01600	0.0360*	
H27A	−0.19780	0.59470	0.04030	0.0510*	
H27B	−0.05360	0.55370	0.03330	0.0510*	
H27C	−0.11360	0.66620	0.00500	0.0510*	
H28A	−0.23220	0.75930	0.09200	0.0430*	
H28B	−0.11220	0.83240	0.12060	0.0430*	
H28C	−0.14980	0.83870	0.05920	0.0430*	
H31	0.15910	0.42270	0.12580	0.0350*	
H32	0.32010	0.36810	0.07700	0.0400*	
H34	0.53920	0.63800	0.13190	0.0430*	
H35	0.38040	0.68940	0.18150	0.0350*	
H36A	0.53140	0.30950	0.06570	0.1030*	
H36B	0.46140	0.37640	0.01600	0.1030*	
H36C	0.61790	0.36640	0.02680	0.1030*	

### Atomic displacement parameters (Å^2^)

**Table:**

	*U*^11^	*U*^22^	*U*^33^	*U*^12^	*U*^13^	*U*^23^
O1	0.0324 (6)	0.0370 (7)	0.0197 (6)	0.0040 (5)	−0.0016 (5)	0.0026 (5)
O2	0.0369 (8)	0.0764 (11)	0.0524 (9)	0.0096 (7)	0.0125 (7)	−0.0272 (8)
N1	0.0231 (7)	0.0226 (7)	0.0193 (7)	−0.0005 (5)	0.0006 (5)	−0.0018 (5)
N2	0.0261 (7)	0.0275 (7)	0.0185 (7)	0.0031 (6)	0.0032 (5)	−0.0056 (6)
N3	0.0263 (7)	0.0262 (7)	0.0191 (7)	0.0004 (6)	0.0023 (6)	−0.0022 (6)
N4	0.0183 (6)	0.0238 (7)	0.0238 (7)	0.0013 (5)	0.0029 (5)	0.0001 (6)
C1	0.0265 (8)	0.0230 (8)	0.0240 (8)	0.0003 (7)	−0.0025 (7)	−0.0062 (7)
C2	0.0280 (9)	0.0263 (9)	0.0214 (8)	0.0024 (7)	−0.0017 (7)	−0.0041 (7)
C3	0.0302 (9)	0.0246 (8)	0.0167 (8)	0.0041 (7)	−0.0029 (6)	−0.0010 (6)
C4	0.0249 (8)	0.0299 (9)	0.0218 (8)	0.0063 (7)	0.0034 (7)	−0.0011 (7)
C5	0.0252 (9)	0.0344 (10)	0.0258 (9)	−0.0004 (7)	0.0069 (7)	−0.0030 (7)
C6	0.0312 (9)	0.0286 (9)	0.0235 (9)	−0.0054 (7)	0.0072 (7)	−0.0011 (7)
C7	0.0317 (9)	0.0209 (8)	0.0246 (9)	0.0003 (7)	0.0022 (7)	0.0027 (7)
C8	0.0294 (9)	0.0235 (9)	0.0248 (9)	0.0037 (7)	0.0016 (7)	−0.0005 (7)
C9	0.0246 (8)	0.0233 (8)	0.0251 (8)	0.0044 (6)	0.0017 (7)	−0.0021 (7)
C10	0.0243 (8)	0.0229 (8)	0.0218 (8)	−0.0018 (6)	0.0061 (6)	−0.0042 (6)
C11	0.0253 (8)	0.0240 (9)	0.0213 (8)	−0.0026 (7)	0.0032 (6)	−0.0011 (7)
C12	0.0245 (8)	0.0242 (9)	0.0213 (8)	−0.0029 (6)	0.0043 (6)	0.0020 (6)
C13	0.0253 (9)	0.0263 (9)	0.0324 (9)	−0.0053 (7)	0.0049 (7)	−0.0018 (7)
C14	0.0286 (9)	0.0269 (9)	0.0344 (10)	−0.0055 (7)	−0.0013 (7)	−0.0040 (8)
C15	0.0313 (9)	0.0249 (9)	0.0279 (9)	−0.0021 (7)	−0.0034 (7)	−0.0005 (7)
C16	0.0452 (11)	0.0346 (10)	0.0260 (9)	−0.0029 (8)	0.0114 (8)	−0.0014 (8)
C17	0.0219 (8)	0.0280 (9)	0.0359 (10)	−0.0022 (7)	0.0072 (7)	−0.0056 (7)
C18	0.0226 (8)	0.0276 (9)	0.0358 (10)	0.0047 (7)	−0.0007 (7)	−0.0057 (8)
C19	0.0362 (10)	0.0335 (10)	0.0335 (10)	0.0100 (8)	0.0031 (8)	0.0022 (8)
C20	0.0350 (10)	0.0261 (9)	0.0316 (9)	0.0012 (7)	0.0062 (8)	−0.0026 (7)
C21	0.0395 (10)	0.0329 (10)	0.0206 (9)	0.0040 (8)	−0.0009 (7)	0.0039 (7)
C22	0.0447 (11)	0.0330 (10)	0.0234 (9)	0.0016 (8)	0.0079 (8)	0.0022 (7)
C23	0.0262 (9)	0.0362 (10)	0.0325 (10)	0.0019 (7)	−0.0010 (7)	−0.0056 (8)
C24	0.0326 (10)	0.0535 (13)	0.0407 (11)	−0.0045 (9)	0.0139 (8)	−0.0092 (9)
C25	0.0326 (9)	0.0353 (10)	0.0269 (9)	0.0059 (8)	0.0084 (7)	−0.0044 (8)
C26	0.0395 (10)	0.0283 (9)	0.0221 (9)	0.0047 (8)	0.0029 (7)	−0.0066 (7)
C27	0.0366 (10)	0.0365 (10)	0.0260 (9)	0.0009 (8)	−0.0047 (7)	−0.0080 (8)
C28	0.0295 (9)	0.0296 (10)	0.0255 (9)	0.0043 (7)	−0.0016 (7)	0.0000 (7)
C29	0.0263 (8)	0.0195 (8)	0.0235 (9)	−0.0004 (6)	0.0007 (7)	0.0003 (6)
C30	0.0264 (8)	0.0233 (8)	0.0208 (8)	0.0046 (7)	−0.0015 (6)	0.0032 (7)
C31	0.0317 (9)	0.0258 (9)	0.0294 (9)	0.0020 (7)	0.0004 (7)	0.0000 (7)
C32	0.0380 (10)	0.0311 (9)	0.0281 (9)	0.0110 (8)	−0.0023 (8)	−0.0077 (8)
C33	0.0280 (9)	0.0461 (11)	0.0290 (10)	0.0137 (8)	0.0023 (7)	−0.0056 (8)
C34	0.0249 (9)	0.0429 (11)	0.0396 (11)	0.0006 (8)	0.0032 (8)	−0.0080 (9)
C35	0.0265 (9)	0.0312 (9)	0.0292 (9)	0.0032 (7)	−0.0013 (7)	−0.0064 (7)
C36	0.0559 (14)	0.0877 (19)	0.0634 (16)	0.0183 (13)	0.0151 (12)	−0.0374 (14)

### Geometric parameters (Å, º)

**Table:**

O1—C29	1.2343 (19)	C34—C35	1.380 (2)
O2—C33	1.374 (2)	C1—H1	1.0000
O2—C36	1.420 (3)	C6—H6	1.0000
N1—C1	1.494 (2)	C7—H7	1.0000
N1—C12	1.501 (2)	C12—H12	1.0000
N1—C29	1.376 (2)	C13—H13A	0.9900
N2—C3	1.380 (2)	C13—H13B	0.9900
N2—C4	1.380 (2)	C14—H14A	0.9900
N3—C6	1.483 (2)	C14—H14B	0.9900
N3—C7	1.474 (2)	C15—H15A	0.9800
N4—C9	1.377 (2)	C15—H15B	0.9800
N4—C10	1.378 (2)	C15—H15C	0.9800
N2—H2A	0.8800	C16—H16A	0.9800
N3—H3N	0.924 (18)	C16—H16B	0.9800
N4—H4A	0.8800	C16—H16C	0.9800
C1—C2	1.561 (2)	C17—H17	0.9500
C1—C14	1.538 (2)	C18—H18	0.9500
C2—C3	1.518 (2)	C19—H19A	0.9800
C2—C27	1.546 (2)	C19—H19B	0.9800
C2—C28	1.531 (2)	C19—H19C	0.9800
C3—C26	1.372 (2)	C20—H20A	0.9800
C4—C5	1.510 (2)	C20—H20B	0.9800
C4—C25	1.369 (2)	C20—H20C	0.9800
C5—C23	1.539 (2)	C21—H21A	0.9900
C5—C6	1.547 (2)	C21—H21B	0.9900
C5—C24	1.542 (2)	C22—H22A	0.9900
C6—C22	1.547 (2)	C22—H22B	0.9900
C7—C8	1.546 (2)	C23—H23A	0.9800
C7—C21	1.523 (2)	C23—H23B	0.9800
C8—C20	1.549 (2)	C23—H23C	0.9800
C8—C9	1.516 (2)	C24—H24A	0.9800
C8—C19	1.540 (2)	C24—H24B	0.9800
C9—C18	1.370 (2)	C24—H24C	0.9800
C10—C11	1.517 (2)	C25—H25	0.9500
C10—C17	1.375 (2)	C26—H26	0.9500
C11—C16	1.549 (2)	C27—H27A	0.9800
C11—C12	1.573 (2)	C27—H27B	0.9800
C11—C15	1.531 (2)	C27—H27C	0.9800
C12—C13	1.536 (2)	C28—H28A	0.9800
C13—C14	1.527 (3)	C28—H28B	0.9800
C17—C18	1.411 (2)	C28—H28C	0.9800
C21—C22	1.530 (2)	C31—H31	0.9500
C25—C26	1.410 (3)	C32—H32	0.9500
C29—C30	1.508 (2)	C34—H34	0.9500
C30—C35	1.390 (2)	C35—H35	0.9500
C30—C31	1.390 (2)	C36—H36A	0.9800
C31—C32	1.390 (2)	C36—H36B	0.9800
C32—C33	1.383 (3)	C36—H36C	0.9800
C33—C34	1.386 (3)		
			
C33—O2—C36	117.72 (16)	C11—C12—H12	107.00
C1—N1—C12	112.15 (12)	C13—C12—H12	107.00
C1—N1—C29	119.01 (13)	C12—C13—H13A	111.00
C12—N1—C29	116.83 (12)	C12—C13—H13B	111.00
C3—N2—C4	110.65 (13)	C14—C13—H13A	111.00
C6—N3—C7	105.81 (12)	C14—C13—H13B	111.00
C9—N4—C10	111.22 (13)	H13A—C13—H13B	109.00
C4—N2—H2A	125.00	C1—C14—H14A	111.00
C3—N2—H2A	125.00	C1—C14—H14B	111.00
C6—N3—H3N	111.0 (11)	C13—C14—H14A	111.00
C7—N3—H3N	111.9 (11)	C13—C14—H14B	111.00
C10—N4—H4A	124.00	H14A—C14—H14B	109.00
C9—N4—H4A	124.00	C11—C15—H15A	109.00
N1—C1—C14	101.34 (12)	C11—C15—H15B	109.00
N1—C1—C2	117.02 (13)	C11—C15—H15C	109.00
C2—C1—C14	117.27 (13)	H15A—C15—H15B	109.00
C1—C2—C28	113.95 (13)	H15A—C15—H15C	110.00
C1—C2—C3	110.60 (13)	H15B—C15—H15C	109.00
C1—C2—C27	105.44 (13)	C11—C16—H16A	109.00
C27—C2—C28	108.30 (13)	C11—C16—H16B	109.00
C3—C2—C27	108.06 (13)	C11—C16—H16C	110.00
C3—C2—C28	110.20 (13)	H16A—C16—H16B	109.00
C2—C3—C26	130.52 (15)	H16A—C16—H16C	109.00
N2—C3—C26	106.43 (14)	H16B—C16—H16C	109.00
N2—C3—C2	123.03 (14)	C10—C17—H17	126.00
C5—C4—C25	130.40 (15)	C18—C17—H17	126.00
N2—C4—C5	123.19 (14)	C9—C18—H18	126.00
N2—C4—C25	106.37 (14)	C17—C18—H18	126.00
C4—C5—C6	111.30 (13)	C8—C19—H19A	109.00
C6—C5—C23	109.22 (13)	C8—C19—H19B	109.00
C4—C5—C23	111.77 (14)	C8—C19—H19C	110.00
C4—C5—C24	108.75 (14)	H19A—C19—H19B	110.00
C6—C5—C24	107.21 (14)	H19A—C19—H19C	109.00
C23—C5—C24	108.44 (14)	H19B—C19—H19C	109.00
N3—C6—C22	104.00 (13)	C8—C20—H20A	109.00
N3—C6—C5	113.18 (13)	C8—C20—H20B	110.00
C5—C6—C22	114.39 (13)	C8—C20—H20C	109.00
N3—C7—C8	114.92 (13)	H20A—C20—H20B	110.00
N3—C7—C21	100.76 (13)	H20A—C20—H20C	110.00
C8—C7—C21	118.57 (14)	H20B—C20—H20C	109.00
C7—C8—C20	108.51 (13)	C7—C21—H21A	111.00
C9—C8—C19	109.67 (13)	C7—C21—H21B	111.00
C7—C8—C9	114.89 (13)	C22—C21—H21A	111.00
C7—C8—C19	107.19 (13)	C22—C21—H21B	111.00
C9—C8—C20	107.94 (13)	H21A—C21—H21B	109.00
C19—C8—C20	108.49 (14)	C6—C22—H22A	111.00
N4—C9—C8	122.37 (14)	C6—C22—H22B	111.00
N4—C9—C18	106.23 (14)	C21—C22—H22A	111.00
C8—C9—C18	130.20 (15)	C21—C22—H22B	111.00
N4—C10—C17	105.94 (14)	H22A—C22—H22B	109.00
N4—C10—C11	122.17 (13)	C5—C23—H23A	110.00
C11—C10—C17	131.82 (15)	C5—C23—H23B	110.00
C12—C11—C16	105.33 (13)	C5—C23—H23C	110.00
C10—C11—C15	109.30 (13)	H23A—C23—H23B	109.00
C15—C11—C16	107.95 (13)	H23A—C23—H23C	109.00
C10—C11—C12	115.78 (13)	H23B—C23—H23C	109.00
C10—C11—C16	107.19 (13)	C5—C24—H24A	109.00
C12—C11—C15	110.90 (13)	C5—C24—H24B	109.00
N1—C12—C13	104.01 (12)	C5—C24—H24C	109.00
N1—C12—C11	116.90 (13)	H24A—C24—H24B	110.00
C11—C12—C13	115.38 (13)	H24A—C24—H24C	109.00
C12—C13—C14	104.81 (13)	H24B—C24—H24C	109.00
C1—C14—C13	105.49 (14)	C4—C25—H25	126.00
C10—C17—C18	108.32 (15)	C26—C25—H25	126.00
C9—C18—C17	108.28 (15)	C3—C26—H26	126.00
C7—C21—C22	102.13 (13)	C25—C26—H26	126.00
C6—C22—C21	105.24 (13)	C2—C27—H27A	109.00
C4—C25—C26	108.45 (15)	C2—C27—H27B	110.00
C3—C26—C25	108.11 (15)	C2—C27—H27C	110.00
O1—C29—N1	121.72 (14)	H27A—C27—H27B	109.00
N1—C29—C30	120.64 (13)	H27A—C27—H27C	110.00
O1—C29—C30	117.41 (14)	H27B—C27—H27C	109.00
C31—C30—C35	117.79 (15)	C2—C28—H28A	109.00
C29—C30—C35	115.71 (14)	C2—C28—H28B	109.00
C29—C30—C31	125.98 (14)	C2—C28—H28C	109.00
C30—C31—C32	121.51 (16)	H28A—C28—H28B	110.00
C31—C32—C33	119.49 (16)	H28A—C28—H28C	109.00
O2—C33—C32	125.09 (17)	H28B—C28—H28C	109.00
O2—C33—C34	115.15 (16)	C30—C31—H31	119.00
C32—C33—C34	119.75 (17)	C32—C31—H31	119.00
C33—C34—C35	120.14 (17)	C31—C32—H32	120.00
C30—C35—C34	121.29 (16)	C33—C32—H32	120.00
N1—C1—H1	107.00	C33—C34—H34	120.00
C2—C1—H1	107.00	C35—C34—H34	120.00
C14—C1—H1	107.00	C30—C35—H35	119.00
N3—C6—H6	108.00	C34—C35—H35	119.00
C5—C6—H6	108.00	O2—C36—H36A	109.00
C22—C6—H6	108.00	O2—C36—H36B	109.00
N3—C7—H7	107.00	O2—C36—H36C	109.00
C8—C7—H7	107.00	H36A—C36—H36B	109.00
C21—C7—H7	107.00	H36A—C36—H36C	109.00
N1—C12—H12	107.00	H36B—C36—H36C	110.00
			
C36—O2—C33—C32	−4.9 (3)	C24—C5—C6—N3	−167.61 (13)
C36—O2—C33—C34	174.72 (18)	C24—C5—C6—C22	73.50 (17)
C12—N1—C1—C2	107.23 (15)	N3—C6—C22—C21	0.44 (17)
C12—N1—C1—C14	−21.59 (16)	C5—C6—C22—C21	124.39 (15)
C29—N1—C1—C2	−111.23 (16)	N3—C7—C8—C9	−48.77 (18)
C29—N1—C1—C14	119.95 (14)	N3—C7—C8—C19	−170.90 (13)
C1—N1—C12—C11	−127.09 (14)	N3—C7—C8—C20	72.12 (17)
C1—N1—C12—C13	1.36 (16)	C21—C7—C8—C9	70.41 (19)
C29—N1—C12—C11	90.47 (17)	C21—C7—C8—C19	−51.73 (19)
C29—N1—C12—C13	−141.08 (14)	C21—C7—C8—C20	−168.70 (14)
C1—N1—C29—O1	−149.49 (15)	N3—C7—C21—C22	−43.89 (15)
C1—N1—C29—C30	36.2 (2)	C8—C7—C21—C22	−170.18 (14)
C12—N1—C29—O1	−9.7 (2)	C7—C8—C9—N4	52.8 (2)
C12—N1—C29—C30	175.94 (13)	C7—C8—C9—C18	−141.52 (18)
C4—N2—C3—C2	−177.68 (14)	C19—C8—C9—N4	173.56 (14)
C4—N2—C3—C26	0.71 (18)	C19—C8—C9—C18	−20.7 (2)
C3—N2—C4—C5	177.31 (14)	C20—C8—C9—N4	−68.43 (19)
C3—N2—C4—C25	−0.66 (18)	C20—C8—C9—C18	97.3 (2)
C7—N3—C6—C5	−153.56 (13)	N4—C9—C18—C17	0.74 (19)
C7—N3—C6—C22	−28.83 (16)	C8—C9—C18—C17	−166.71 (16)
C6—N3—C7—C8	174.58 (13)	N4—C10—C11—C12	−109.38 (16)
C6—N3—C7—C21	45.89 (15)	N4—C10—C11—C15	16.7 (2)
C10—N4—C9—C8	168.50 (14)	N4—C10—C11—C16	133.45 (15)
C10—N4—C9—C18	−0.16 (18)	C17—C10—C11—C12	74.0 (2)
C9—N4—C10—C11	−177.86 (14)	C17—C10—C11—C15	−159.91 (17)
C9—N4—C10—C17	−0.49 (18)	C17—C10—C11—C16	−43.2 (2)
N1—C1—C2—C3	52.15 (18)	N4—C10—C17—C18	0.94 (19)
N1—C1—C2—C27	168.72 (13)	C11—C10—C17—C18	177.95 (16)
N1—C1—C2—C28	−72.64 (18)	C10—C11—C12—N1	75.88 (17)
C14—C1—C2—C3	172.90 (14)	C10—C11—C12—C13	−46.87 (19)
C14—C1—C2—C27	−70.53 (17)	C15—C11—C12—N1	−49.38 (18)
C14—C1—C2—C28	48.11 (19)	C15—C11—C12—C13	−172.14 (14)
N1—C1—C14—C13	33.50 (16)	C16—C11—C12—N1	−165.92 (13)
C2—C1—C14—C13	−95.17 (16)	C16—C11—C12—C13	71.33 (17)
C1—C2—C3—N2	−83.99 (18)	N1—C12—C13—C14	19.90 (16)
C1—C2—C3—C26	98.0 (2)	C11—C12—C13—C14	149.28 (13)
C27—C2—C3—N2	161.07 (15)	C12—C13—C14—C1	−33.92 (17)
C27—C2—C3—C26	−16.9 (2)	C10—C17—C18—C9	−1.1 (2)
C28—C2—C3—N2	42.9 (2)	C7—C21—C22—C6	26.63 (17)
C28—C2—C3—C26	−135.07 (18)	C4—C25—C26—C3	0.09 (19)
N2—C3—C26—C25	−0.48 (18)	O1—C29—C30—C31	108.99 (19)
C2—C3—C26—C25	177.74 (16)	O1—C29—C30—C35	−62.4 (2)
N2—C4—C5—C6	−53.7 (2)	N1—C29—C30—C31	−76.4 (2)
N2—C4—C5—C23	68.7 (2)	N1—C29—C30—C35	112.15 (17)
N2—C4—C5—C24	−171.63 (15)	C29—C30—C31—C32	−171.98 (16)
C25—C4—C5—C6	123.70 (19)	C35—C30—C31—C32	−0.7 (2)
C25—C4—C5—C23	−113.9 (2)	C29—C30—C35—C34	173.61 (16)
C25—C4—C5—C24	5.8 (2)	C31—C30—C35—C34	1.4 (3)
N2—C4—C25—C26	0.34 (19)	C30—C31—C32—C33	−0.5 (3)
C5—C4—C25—C26	−177.43 (16)	C31—C32—C33—O2	−179.42 (17)
C4—C5—C6—N3	73.58 (16)	C31—C32—C33—C34	1.0 (3)
C4—C5—C6—C22	−45.31 (18)	O2—C33—C34—C35	−179.91 (18)
C23—C5—C6—N3	−50.31 (18)	C32—C33—C34—C35	−0.3 (3)
C23—C5—C6—C22	−169.19 (14)	C33—C34—C35—C30	−1.0 (3)

### Hydrogen-bond geometry (Å, º)

Cg1 is the centroid of the pyrrole ring N2/C3/C4/C25/C26 and *Cg*2 is the centroid of the benzene ring C30–C35.

**Table:**

*D*—H···*A*	*D*—H	H···*A*	*D*···*A*	*D*—H···*A*
N2—H2*A*···N3	0.88	2.57	3.0671 (18)	117
N4—H4*A*···N3	0.88	2.33	2.8759 (18)	121
C28—H28*B*···N4	0.98	2.59	3.561 (2)	171
C28—H28*B*···*Cg*1	0.98	2.45	3.3632 (18)	155
N3—H3*N*···O1^i^	0.924 (18)	2.283 (18)	3.1401 (17)	154.0 (15)
C20—H20*B*···O1^i^	0.98	2.56	3.530 (2)	170
C15—H15*A*···*Cg*2^i^	0.98	2.85	3.7176 (19)	148

Symmetry code: (i) −*x*+1/2, *y*+1/2, −*z*+1/2.
